# Manganese oxide synthesized from spent Zn-C battery for supercapacitor electrode application

**DOI:** 10.1038/s41598-019-44778-z

**Published:** 2019-06-20

**Authors:** Rifat Farzana, Kamrul Hassan, Veena Sahajwalla

**Affiliations:** Centre for Sustainable Materials Research and Technology (SMaRT@UNSW), School of Materials Science and Engineering, UNSW Sydney, Sydney, NSW 2052 Australia

**Keywords:** Synthesis and processing, Materials for devices

## Abstract

Manganese oxide (Mn_3_O_4_) nanomaterials have promising potential to be used as supercapacitor electrode materials due to its high energy storage performance and environmental compatibility. Besides, every year huge volume of waste batteries including Zn-C battery ends up in landfill, which aggravates the burden of waste disposal in landfill and creates environmental and health threat. Thus, transformation of waste battery back into energy application, is of great significance for sustainable strategies. Compared with complex chemical routes which mostly apply toxic acids to recover materials from Zn-C battery, this study establishes the recovery of Mn_3_O_4_ particles via thermal route within 900 °C under controlled atmosphere. Synthesized Mn_3_O_4_ were confirmed by XRD, EDS, FTIR, XPS and Raman analysis and FESEM micrographs confirmed the coexistence of spherical and cubic Mn_3_O_4_ particles. Mn_3_O_4_ electrode derived from waste Zn-C battery demonstrate compatible electrochemical performance with standard materials and conventional synthesis techniques. Mn_3_O_4_ electrode exhibited highest capacitance value of 125 Fg^−1^ at 5 mVs^−1^ scan rate. The stability of the electrode showed good retention in discharge and charge capacity by about 80% after 2100 cycles. This study demonstrates that waste Zn-C battery can be further utilized for energy storage application, providing sustainable and economic benefits.

## Introduction

Supercapacitor become more attractive and efficient energy storage and conversion devices than batteries due to high specific power, long life cycle and fast charge-discharge rate^1^. Hence, supercapacitors are undergoing rapid development with widespread application in automobiles, electronics and in industries^1^. Supercapacitors mainly consist of electrodes, current collectors, electrolyte and a spacer but electrodes are the key element of supercapacitor’s performance^1–3^. In general supercapacitor store energy via either electrical double layer capacitance (EDLC) principle or the pseudo-capacitance mechanism. Energy density in EDLC is managed by the electrostatic capability of the absorbing electrolytes (anions, cations), by active materials embedded within electrodes and therefore carbon materials such as, activated carbon, carbon nanotubes etc. with high surface area are used. Besides, in pseudo-capacitance mechanism, energy density is governed by reversible redox interactions of the active materials as electrode and generally transition metal oxides and conducting polymers are used as active materials^1,4,5^. Nanostructured metal oxides as electrode material, have attracted attention due to design flexibility, low resistance and high specific capacitance^6^. Manganese oxide (MnO_x_) has a wide range of applications including catalysis, electrochemical materials, high-density magnetic storage media etc. Recently, MnOx materials including Mn_3_O_4_ were substantially reported as supercapacitor electrode materials due to its environmental compatibility, low cost and good electrochemical performance compared to other oxides like ruthenium oxide^7^. Mn_3_O_4_ materials for different application covers a wide range of synthesis routes including reduction, thermal decomposition, coprecipitation, hydrothermal, sol-gel^8–10^ etc. using reagent grade materials. Besides, Mn_3_O_4_/Mn_3_O_4_-composite materials for supercapacitor application includes electrostatic spray deposition, hydrothermal synthesis etc. techniques^11–13^. However, the synthesis routes and preparation techniques are complex and may use toxic acid. The use of waste carbonaceous materials like bio-waste, polymers etc. have been reported for energy storage applications, but metal oxides from waste were ignored. To best our knowledge, no study has reported Mn_3_O_4_ from waste materials/waste battery for supercapacitor application. Therefore, a facile thermal nanosizing technique, to recover value-added materials from waste battery for supercapacitor application will be a perceptibly attractive and economically viable solution. Simultaneously the approach will divert hazardous battery waste from landfill.

Batteries become an integral part of portable power solution of modern lifestyle. It is estimated that about 350 million handheld (less than 1 kg in weight) batteries are consumed in Australia every year^14^. Primary/non-rechargeable/single-use batteries contribute 81% of handheld batteries and zinc-carbon battery (Zn-C) is one of them which comprises 19% on proportion count and mostly sent to landfill. Used batteries are hazardous as defined by the Federal Department of Environment and therefore they require an export permit under the Hazardous Waste Act^14,15^. Although batteries are potential mineral resources, almost 95% (equivalent to 8,000 tonnes) end up in landfill in Australia each year^14,16^. In landfill, the chemicals inside batteries leach and cause environmental and human health threat by polluting land and water^16,17^. Under the Australian standard for electronic waste recycling (AS/NZS 5377:2013)^18^ and other countries (European Commission, Directive 2006/66)^19^, disposal of used batteries to landfill is not also an acceptable processing option. Therefore, it is vital to identify sustainable and viable solutions to recover value-added materials from waste batteries for different applications. Several studies on mechanical separation methods followed by pyrometallurgical^20–22^ or hydrometallurgical^23–29^ or bio-hydrometallurgical^30,31^ treatments are reported to extract value-added content from spent Zn-C batteries. However, proposed applications of the recovered metal/materials were not considered in most of the cited studies so far. So, avoiding complex hydrometallurgical route for the metal separation, this study investigated the electrochemical performance of manganese oxide (Mn_3_O_4_) nanoparticles (NPs) for supercapacitor application derived from waste Zn-C battery via simple thermal route.

Our research group has conducted studies on the fastest growing e-waste, focusing on thermal transformation of waste into value-added materials. Recently, activated carbon, derived from waste CD for supercapacitor application is reported^32^. To best our knowledge, this study for the first time reports the performance of Mn_3_O_4,_ derived from waste Zn-C battery for supercapacitor application. A facile thermal transformation study under inert (at 900 °C) and air (at 800 °C) atmosphere is investigated to synthesise Mn_3_O_4_ NPs from spent Zn-C battery. Mn_3_O_4_ NPs were confirmed by XRD, FTIR, Raman, EDS and XPS analysis. The morphological studies and structure of Mn_3_O_4_ NPs were analysed by using SEM, TEM, SAED techniques. Electrochemical analysis was conducted by cyclic voltammetry, galvanostatic charge-discharge and electrochemical impedance spectroscopy to assess the capacitance properties of as synthesised Mn_3_O_4_ NPs. The specific capacitance of 125 Fg^−1^ at the scan rate 10 mVs^−1^ was achieved with good cycle stability. As mining is energy-sensitive and non-sustainable, waste Zn-C battery as an urban mining resource for energy application will be a promising and sustainable solution for future.

## Results and Discussion

### Characterization of waste battery

**Figure 1:**
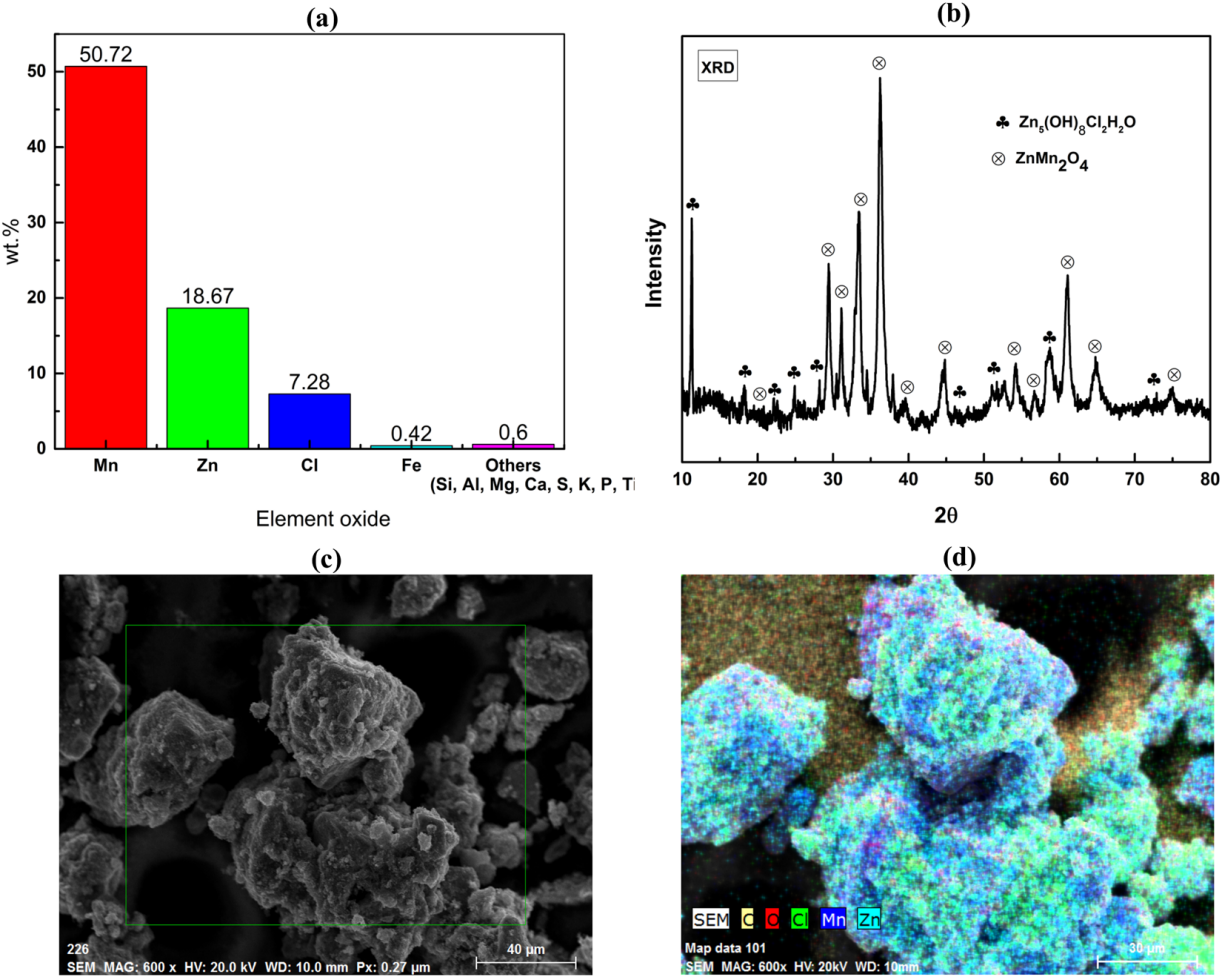
(**a**) Elemental analysis, (**b**) XRD, (**c**) SEM and (**d**) EDS mapping of waste Zn-C battery.

In Zn-C battery, black powdered material (used for this study) contains manganese dioxide (MnO_2_) and carbon which act as cathode and is wetted with electrolyte, zinc chloride (ZnCl_2_). Spent battery black powder composition by XRF analysis showed highest concentration of manganese oxide and substantial amount of zinc oxide and chlorine (Cl) (Fig. 1a). Presence of minor elemental oxide includes Fe, Si, Mg, Ca, S, K, P etc. and loss of ignition (LOI) value was around 22%.

XRD diffraction peaks of spent battery powder shown in Fig. 1b confirmed the presence of mainly ZnMn_2_O_4_ (hetaerolite) and Zn_5_(OH)_8_Cl_2_H_2_O (simonkolleite) phases which formed due to the electro-chemical reaction within cathode and anode over time. Formation of these compounds is in agreement with presence major elements Zn, Mn, Cl and moisture in waste battery powder.

SEM image shown in Fig. 1c confirmed that the morphology of the spent battery powder is in agglomerated state without certain orientation or shape. Particle sizes include both coarse and fine particles and could not be distinguished clearly but both coarse and fine particles co-exist. EDS mapping in Fig. 1d, showed that aggregated particles contains Mn, Zn, Cl and O. Presence of these elements are also in agreement with XRF analysis and XRD results. Presence of C peak can be attributed to the carbon present in cathode and/or carbon from the carbon rod while dismantling.

Thermogravimetric analysis along with DTG profile of spent battery powder at a rate of 20 °C/min from room temperature to 1200 °C under N_2_ atmosphere is shown in Fig. S1 (SI). It was evident from the TGA/DTG profile that gradual weight loss occurred between 100 to 800 °C. Initial weight loss below 200 °C was attributed to the moisture loss and below 600 °C due to decomposition of ZnMn_2_O_4_ into MnO and ZnO (reaction 1) and Zn_5_(OH)_8_Cl_2_H_2_O into Zn(OH)Cl and ZnO (reaction 2). Major weight loss after 800 °C is attributed to ZnO evaporation via Zn vapor which is discussed in details in our previous study^33^. A total weight loss of ~52 wt.% was observed in the TGA data. Though dominant weight loss temperature from the DTG graph was 1120 °C, lower temperature 900 °C was considered for this study to reduce energy consumption.

### Characterization of synthesised Mn_3_O_4_

Zn_5_(OH)_8_Cl_2_H_2_O decomposes to ZnO/ZnO bearing compound as per reaction 1 even below 900 °C^34^ and evaporated from the residue via formation of Zn vapor which is discussed in previous study^33^. ZnMn_2_O_4_ also started to transform to MnO and ZnO as per the reaction 2^35^ where Zn/ZnO is recovered as condensate via Zn vapor leaving behind manganese oxide as residue. Therefore, during the oxidation stage at 800 °C under air atmosphere, residual manganese oxide will transform to Mn_3_O_4_ as per reaction 3. As received Mn_3_O_4_ powder was used for analysis and electrode fabrication.

$$\begin{array}{ll}{{\rm{Zn}}}_{5}{({\rm{OH}})}_{8}{{\rm{Cl}}}_{2}{{\rm{H}}}_{2}{\rm{O}}\to 2{\rm{Zn}}({\rm{OH}}){\rm{Cl}}+3{\rm{ZnO}}+4{{\rm{H}}}_{2}{\rm{O}}\,\uparrow  & {\boldsymbol{reaction}}\,{\bf{1}}\\ {{\rm{ZnMn}}}_{2}{{\rm{O}}}_{4}\to {\rm{ZnO}}+{\rm{MnO}}+0.5{{\rm{O}}}_{2}\,\uparrow  & {\boldsymbol{reaction}}\,{\bf{2}}\\ 6{\rm{MnO}}+{{\rm{O}}}_{2}\to 2{{\rm{Mn}}}_{3}{{\rm{O}}}_{4} & {\boldsymbol{reaction}}\,{\bf{3}}\end{array}$$

**Figure 2:**
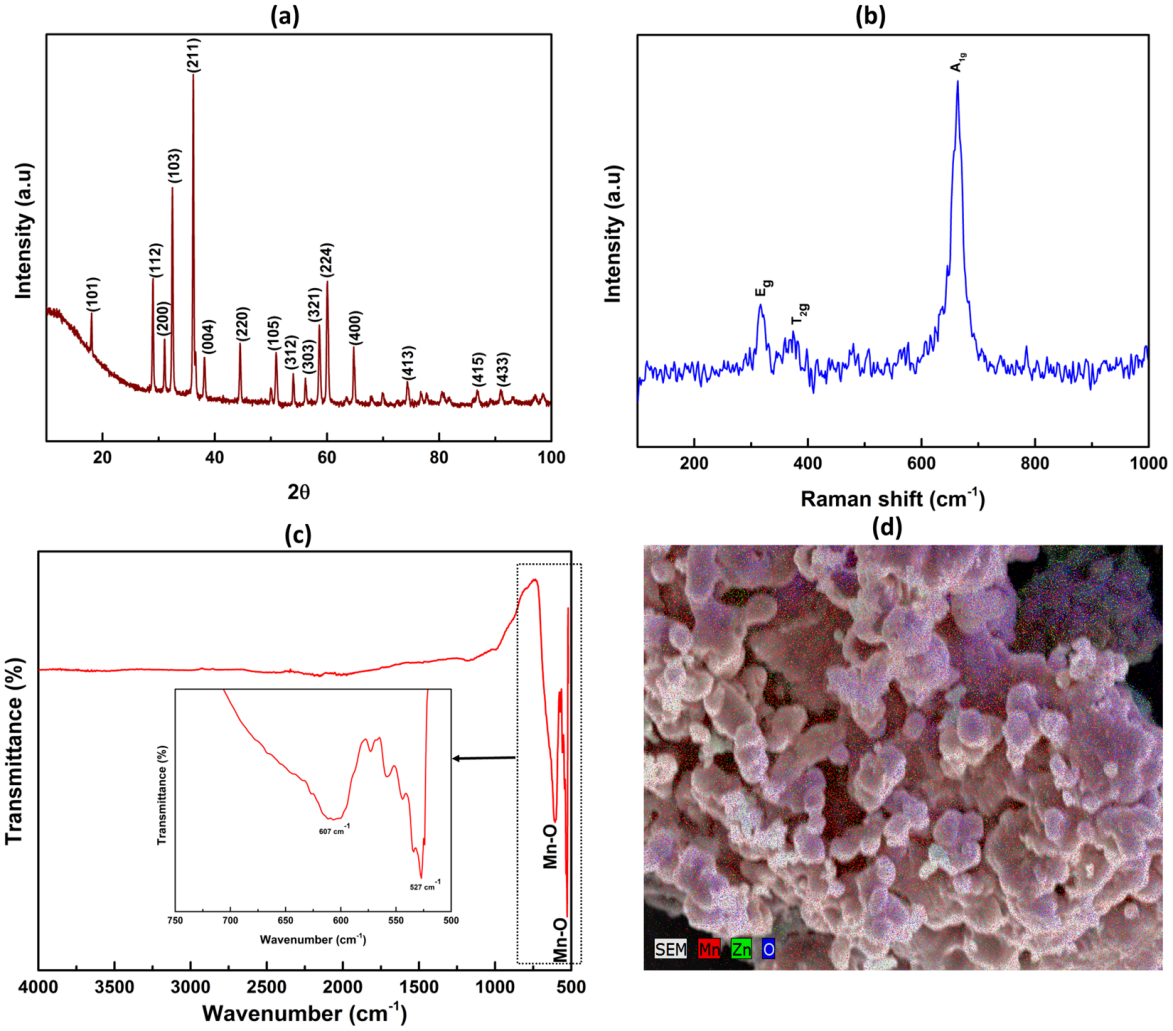
(**a**) XRD, (**b**) Raman (**c**) FTIR spectra (inset showing the zoomed region from 500–750 cm^−1^) and (**d**) EDS mapping of synthesised Mn_3_O_4_ NPs.

The structure and quality of the synthesised Mn_3_O_4_ particles were measured by XRD, FTIR, Raman and EDS analysis and is shown in Fig. 2. XRD pattern of the as synthesised powder (Fig. 2a) assigned to a tetragonal Mn_3_O_4_ (Hausmannite) phase (ICDD Code: 04-006-8183) with space group I41/amd (141)^36^. The diffraction peak positions at 2θ values ~ 18, 29, 31, 32, 36, 38, 44, 51, 59, 60 and 65 were assigned to corresponding crystal planes of (101), (112), (200), (103), (211), (004), (220), (105), (321), (224) and (400) respectively and in harmony with reported literature^36,37^.

The FTIR spectra (Fig. 2b) of as synthesised powder materials, was performed to determine the vibrational transitions of bonds and mainly the characteristic absorption peaks for metal-oxygen bonding (Mn-O) were observed. The peaks at ~527 cm^−1^ and 607 cm^−1^ were assigned to the stretching vibration of the spinel metal oxide (MnO.Mn_2_O_3_)^36^. Peaks at ~527 and 607 cm^−1^ are attributed to Mn^3+^ ions, which occupy the octahedral sites and Mn^2+^ ions which occupies the tetrahedral sites of Mn_3_O_4_ NPs^36–38^.

Raman scattering (Fig. 2c) showed one sharp band at ~663 cm^−1^ along with two weak Raman bands at ~370, 317 cm^−1^. Raman band at ~663, 370, 317 cm^−1^ were assigned to the characteristic A_1g,_ T_2g_ and E_g_ active modes which usually originates from stretching of modes of tetragonal hausmannite with spinal structure and also in agreement in reported literature^7,39,40^.

EDS mapping of Mn_3_O_4_ NPs identified Mn and O were uniformly distributed which also established the formation of Mn_3_O_4_. Minor distribution of Zn peak could be attributed to the residual ZnO which could be removed with longer time or higher temperature. The XRD, FTIR, Raman and EDS results identified that synthesised powder were crystalline Mn_3_O_4_ with hausmannite structure without/minor impurity levels.

**Figure 3:**
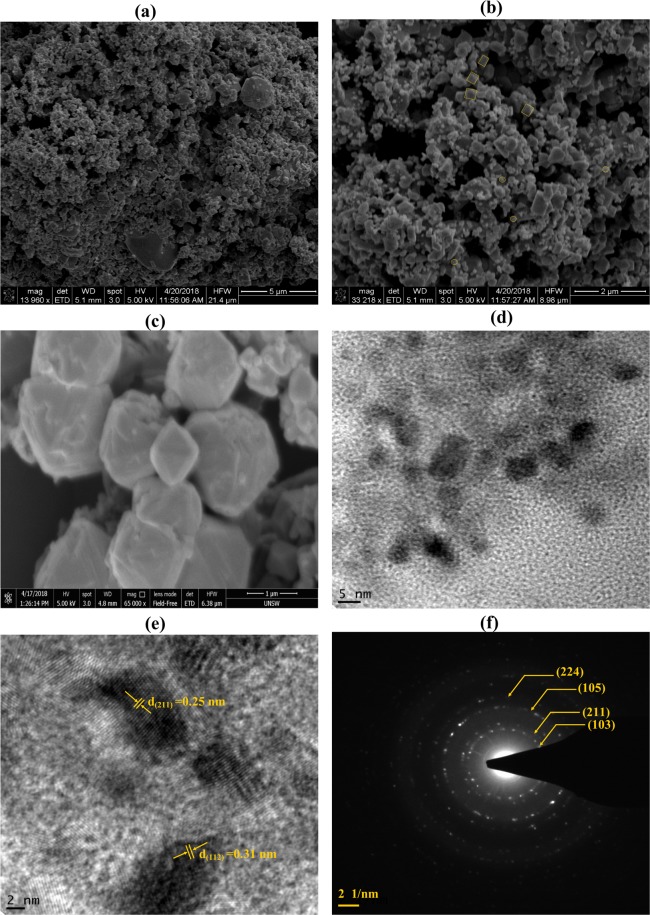
(**a**) Low (**b**,**c**) High magnification FESEM image, (**d**) TEM image, (**e**) Lattice fringes and (**f**) SAED image of synthesised Mn_3_O_4._

The morphology of as prepared Mn_3_O_4_ particles from waste battery were observed by FESEM and TEM images. The low magnification micrograph in Fig. 3a showed mainly spherical particles besides high magnification micrograph (Fig. 3b) showed that the particles were dispersed as either spherical or cubic and some were with uneven shapes. The cubic shape particles are highlighted in the figure. It was observed that the size of the particles was in the nanometer range however some larger particles within ~1 μm were also evident (Fig. 3c). In the HRTEM micrographs (Fig. 3e), the lattice fringes were seen clearly and matched with d-spacing of 0.25 nm and 0.31 nm which are corresponding d-spacing value of (211) and (112) planes of crystalline Mn_3_O_4_ nanoparticles. The SAED pattern (Fig. 3f) showed small spots making up rings coming from the Bragg reflection from each crystallite and confirmed the polycrystalline nature of Mn_3_O_4._ The diffraction rings were well matched with inter planner distance corresponded to (103), (211), (105) and (224) planes of Mn_3_O_4_. The nanoparticle diameter of Mn_3_O_4_ were measured with XRD data using Scherrer equation:

1 $$d=\frac{k\,\lambda }{{\rm{\beta }}\,\cos \,{\rm{\theta }}}$$

where k is the shape factor (0.9), λ is the X-ray wavelength of CuKα radiation (0.154 nm), θ is the Bragg diffraction angle and β is the full width at half maximum of the diffraction peak (in radians). The mean particle diameter calculated using this formula for (211) plane at 2θ = 36° was around 15 nm, which were within the particle diameter range from TEM images.

**Figure 4:**
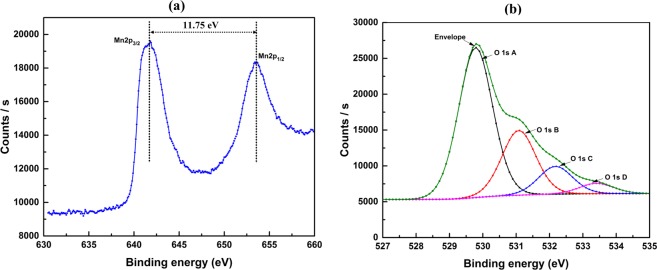
XPS analysis showing the Mn 2p and O 1 s region of synthesised Mn_3_O_4_ NPs.

XPS analysis of the as received powder was conducted with reference to the C1s binding energy (284.8 eV) as an internal standard. Obvious Mn2p and O1s peaks were observed along with low atomic concentration of K, Zn, Ca, Cl as impurity therefore ignored. Figure 4 is showing the high-resolution spectra of Mn2p and O1s to confirm the composition and oxidation state of synthesised Mn_3_O_4_ NPs. It was observed that Mn 2p peak consists of two main spin−orbital lines with binding energies at ~641.65 and 653.4 eV which are attributed to Mn 2p_3/2_ and Mn 2p_1/2_ respectively. The observed difference of binding energy value of ~11.75 eV between the spin-orbit splitting of Mn 2p_3/2_ and Mn 2p_1/2_ levels and their position are in agreement with the reported value for Mn_3_O_4_ in literature^37,40,41^. The highest O1s A peak was observed at 529.7 eV and attributed to the Mn-O and O1s B peak observed at 531.08 eV can be attributed to loosely bonded Mn-OH either due to oxidation or water or adventitious contamination^42^. Low intensity peak of O1s C and D at 532.18 eV, 533.4 eV could be assigned to the residual O^2−^ species bonded with carbon^43^. Though EDS and XPS analysis identified elements like Zn, Ca, Cl etc., these could be removed by selective dissolution using acids to achieve high purity product.

Hydrophilic materials are generally wettable and suitable for the electrode material in aqueous electrode as it can increase availability of the pores and can facilitate ion transport within the electrode. In general, equilibrium contact angle >90°, represents that surface is hydrophobic otherwise hydrophilic^44^. Furthermore, surface roughness and chemical composition are the main features to determine the surface energy and surface energy is inversely proportional to the contact angle^44^. Contact angle image of Mn_3_O_4_ NPs is given in Fig. S2 (SI). Wettability and surface energy of Mn_3_O_4_ NPs were calculated from the contact angle measurement using Young’s equation and Owens and Wendt and that of Wu respectively.

$$\begin{array}{ll}\text{Young}\mbox{'}s\,{\rm{equation}}: & \cos \,{\theta }_{w}={\gamma }_{sv}-\frac{{\gamma }_{sl}}{{\gamma }_{lv}}\\ {\rm{Owens}}\,{\rm{and}}\,{\rm{Wendt}}\,{\rm{equation}}: & {\gamma }_{sl}={\gamma }_{sv}+{\gamma }_{lv}-2(\sqrt{{\gamma }_{sv}^{D}{\gamma }_{lv}^{D}}+\sqrt{{\gamma }_{sv}^{p}{\gamma }_{lv}^{p}})\\ {\rm{Wu}}\,{\rm{equation}}: & \,{\gamma }_{sl}={\gamma }_{sv}+{\gamma }_{lv}-4(\frac{{\gamma }_{lv}^{D}\times {\gamma }_{sv}^{D}}{{\gamma }_{lv}^{D}+{\gamma }_{sv}^{D}}+\frac{{\gamma }_{lv}^{P}\times {\gamma }_{sv}^{P}}{{\gamma }_{lv}^{P}+{\gamma }_{sv}^{P}})\end{array}$$

here, *γ*_*sv*_, *γ*_*lv*_ and *γ*_*sl*_ denote the energies of surface of the solid–vapor, liquid–vapor and solid–liquid interfaces, correspondingly, and *θ*_*w*_ refers the equilibrium contact angle. And ${\gamma }_{lv}^{D}$, ${\gamma }_{sv}^{D}$ and ${\gamma }_{lv}^{p}$,$\,{\gamma }_{sv}^{p}$ are the dispersive and polar components of liquid–vapor (${\gamma }_{lv}$) energy and solid–vapor (${\gamma }_{sv}$) energy, respectively. Synthesised Mn_3_O_4_ NPs showed hydrophilic nature and high surface energy and calculated values are shown in Table 1.

**Table 1:** Contact angles and calculated surface energy of glass, and Mn_3_O_4_/glass.

Sample	Contact angle (°)	Owens–Wendt method			Wu method		
		${{\boldsymbol{\gamma }}}_{{\boldsymbol{sv}}}({\boldsymbol{mN}}/{\boldsymbol{m}})$	${{\boldsymbol{\gamma }}}_{{\boldsymbol{sv}}}^{{\boldsymbol{D}}}({\boldsymbol{mN}}/{\boldsymbol{m}})$	${{\boldsymbol{\gamma }}}_{{\boldsymbol{sv}}}^{{\boldsymbol{p}}}({\boldsymbol{mN}}/{\boldsymbol{m}})$	${{\boldsymbol{\gamma }}}_{{\boldsymbol{sv}}}({\boldsymbol{mN}}/{\boldsymbol{m}})$	${{\boldsymbol{\gamma }}}_{{\boldsymbol{sv}}}^{{\boldsymbol{D}}}({\boldsymbol{mN}}/{\boldsymbol{m}})$	${{\boldsymbol{\gamma }}}_{{\boldsymbol{sv}}}^{{\boldsymbol{p}}}({\boldsymbol{mN}}/{\boldsymbol{m}})$
**Glass**	21	0.0012	0.0012	0.0012	−0.82	−0.207	−0.44
**Mn**_**3**_ **O**_**4**_ **/glass**	39.7	55.59	24.86	30.96	61.99	32.42	29.57

The thermal stability as synthesised Mn_3_O_4_ NPs were investigated by thermal gravimetric analysis is shown in Fig. S3 (SI). A very small weight loss of <2% within (can be attributed to moisture loss) 1200 °C temperature confirmed no further decomposition or weight loss of Mn_3_O_4_ and indicated good thermal stability.

### Electrochemical performance

**Figure 5:**
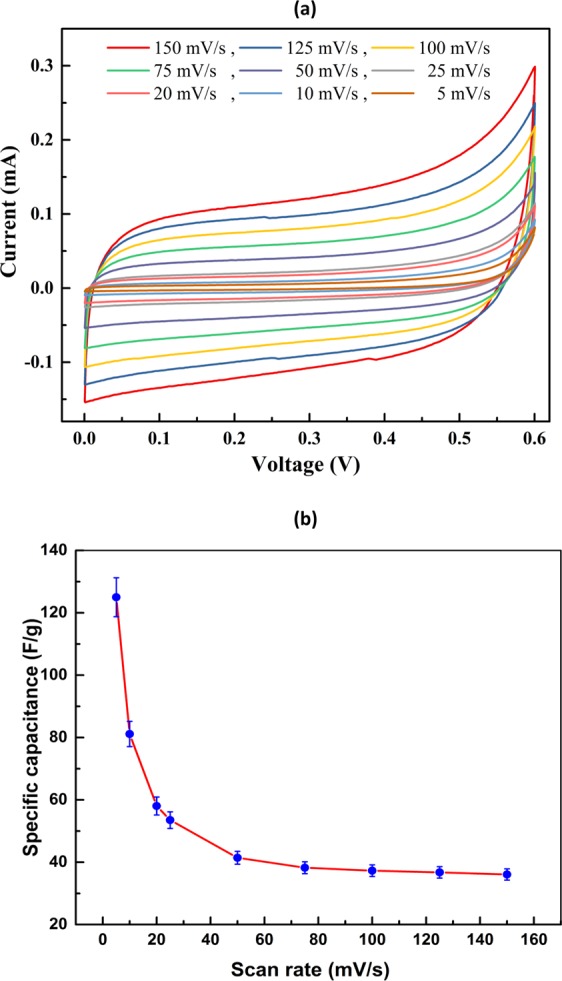
Electrochemical performance of Mn_3_O_4_ electrode in 0.6 M KOH aqueous electrolyte at room temperature (**a**) CV curves at different scan rates from 5 to 150 mV/s in the potential range of 0 to +0.6 V (**b**) specific capacitance as function of different scan rate.

The electrochemical properties of Mn_3_O_4_ NPs were studied using cyclic voltammetry and galvanostatic charge–discharge and cyclic stability measurements. Figure 5 illustrates the cyclic voltammograms of the Mn_3_O_4_ nanoparticles in aqueous 0.6 M KOH as electrolyte at different scan rates of 5–150 mVs^−1^ in the potential range 0 to +0.6 V vs. Hg_2_Cl_2_. From Fig. 5a, it was observed that the electrode showed a pseudo-rectangular-like shape which increased with increasing san rate. This confirmed that the voltametric current was directly proportional to the scan rates of CV, indicating an ideally capacitive behaviour^45^. All the curves were near rectangular shape in nature and showed the mirror image characteristics even at higher scan rates, which could be attributed to the reversible Faradaic redox reactions and electrochemical stability along with high rate performance^4,45^. The specific capacitance of the electrodes was calculated from the respective CV curve using the following equation^4^,

2 $$Csp=\frac{\int IdV}{{\rm{\Delta }}V\times v\times m}$$

Here, *I* (A) is the current, ${\rm{\Delta }}V$ (V) is the potential window, *v* (mVs^−1^) is scan rate and *m* (g) is the mass of active material (Mn_3_O_4_) of electrode. Figure 5b is showing the specific capacitance with different scan rates. The specific capacitance increases with decrease in scan rate and highest specific capacitance of 125 Fg^−1^ was calculated at lowest scan rate of 5 mVs^−1^. At scan rate of 150 mVs^−1^ lowest capacitance 36 Fg^−1^ was measured. The higher specific capacitance at lower scan rate is due to sufficient time for the electrolyte to diffuse on the electrode surface interface. Similar behaviour was observed in other reported literatures^4,46^.

**Figure 6:**
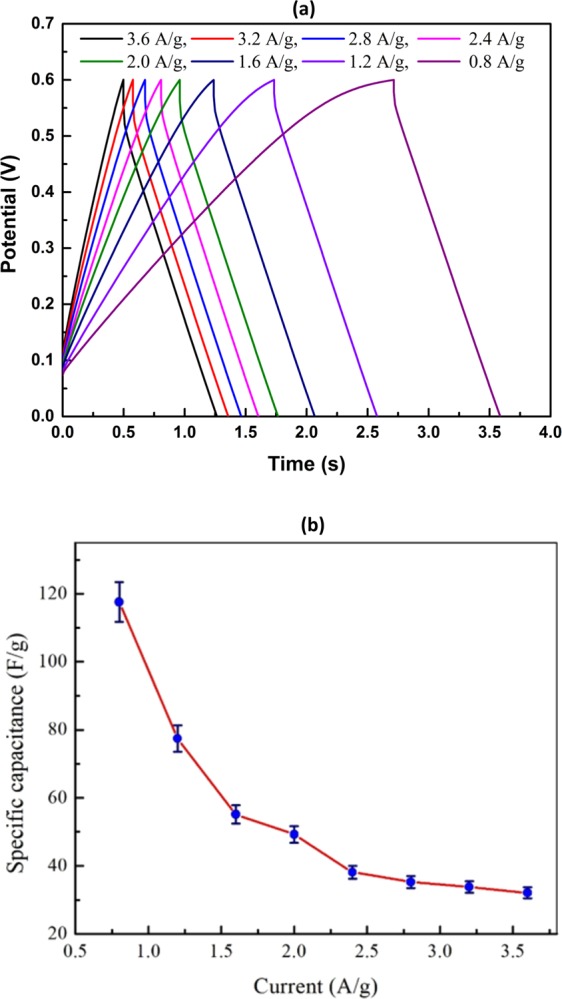
(**a**) Galvanostatic charge-discharge curve of Mn_3_O_4_ NPs in aqueous solution of 0.6 M KOH at different current densities and (**b**) specific capacitance as function of different current densities.

Figure 6a illustrates the galvanostatic charge-discharge (GCD) curve of the Mn_3_O_4_ NPs at different current densities (0.8–3.6 Ag^−1^). From the figure, it is clearly visible that, almost all charge-discharge curves are symmetric in charging counterpart and their corresponding discharge counterparts like triangular charging-discharging characteristics. This might be happened due to the fast charge propagation with an ohmic drop (IR drop) in the conductive ink. The specific capacitance value from the galvanostatic charge-discharge measurement was calculated using the following equation^4^:

3 $${C}_{sp}=\frac{I\,\times \,{\rm{\Delta }}t}{{\rm{\Delta }}V\,\times m}$$

where, I is the discharge current (A), Δt is the discharge time (s), ΔV is the potential window (V) and m is the mass (g) of the active material. Figure 6(b) shows the effect of applied current on specific capacitance of the Mn_3_O_4_ electrode in 0.6 M KOH aqueous electrolyte at room temperature. The decrease in the specific capacitance with increasing current density could be attributed to the diffusion limited process. The highest specific capacitance of 117.56 Fg^−1^ was calculated at lowest current density of 0.8 A/g. At current density of 3.6 A/g lowest capacitance, 32.06 Fg^−1^ was measured. At higher current density, the electrolyte ions do not get adequate time for the diffusion into the inner pores therefore provides lower capacitance^4^.

**Figure 7:**
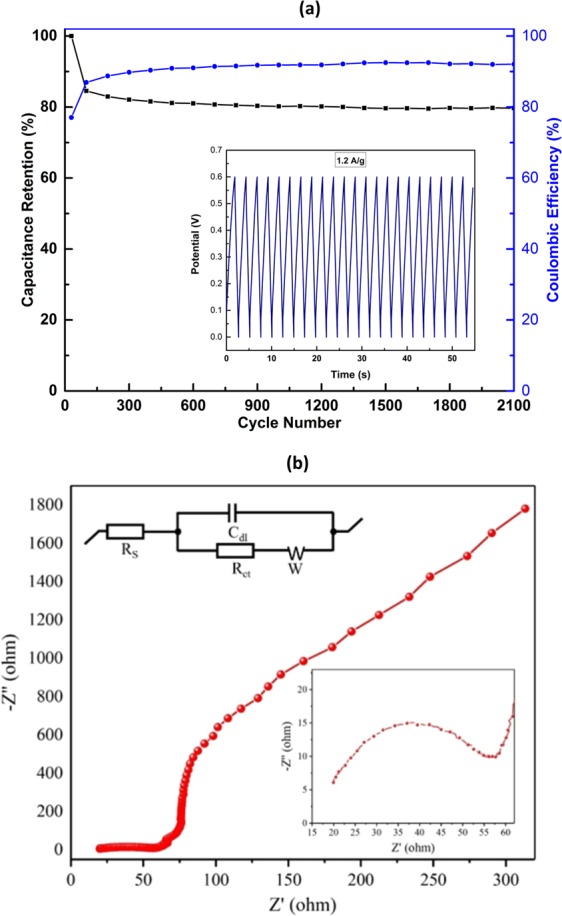
(**a**) Cyclic stability in terms of capacitance retention and coulombic efficiency (inset) Cycling behaviour of Mn_3_O_4_ nanoparticles and (**b**) Electrochemical impedance of Mn_3_O_4_ nanoparticles. Inset shows the pseudo charge transfer resistance in the range of high frequency along with the equivalent circuit.

Besides, for real supercapacitor application the good cycling stability and efficiency is also very important. Figure 7(a) depicts the charge–discharge cyclic stability at a current density of 1.2 Ag^−1^ in 0.6 M KOH as electrolyte and the variation of calculated capacitance retention and coulombic efficiency as a function of cycle number. It was observed that, Mn_3_O_4_ NPs electrode showed good retention in discharge and charge capacity by about ~85% after 100 cycles and ~80% even after 2100 cycles, indicating the long-life cycle of the fabricated electrode with Mn_3_O_4_ NPs synthesised from waste battery. Small decay in capacitance over cycles could occur due to mechanical expansion of conductive ink during the dissolution of Mn in aqueous electrolyte and ion insertion/desertion process in aqueous electrolytes. For metal oxide electrode, poor cyclic stability due to structural damage during the redox process is common^47^. The coulombic/faradic efficiency was measured from ratio of discharging by charging time and demonstrates the practical applicability of electrode material for supercapacitor^48^. The coulombic efficiency increased over time and reached ~92% which validates the improved redox reversibility of electrode material^48^. The GCD curve in Fig. 7(a) (inset) with current density of 1.2 Ag^−1^ showed linear and typical triangular shape which could be attributed to the good electrochemical capacitive characteristic with high degree of reversibility. The retention and coulombic efficiency results validate the long life and good efficiency of the Mn_3_O_4_ electrode derived from waste Zn-C battery.

The capacitive behaviour of the electrode materials and ability to store electrical energy, was measured by electrochemical impedance spectroscopy (EIS) which is one of the authoritative procedures. The typical Nyquist plots of the Mn_3_O_4_ nanoparticles are presented in Fig. 7(b). From the figure it observed that, a straight sloping line in the range of low frequency, which corresponds to the diffusive resistance of the Mn_3_O_4_ nanoparticles. The capacitance value increases at low frequencies due to a larger number of ions moving which cause a decrease in the bulk resistance of the capacitor. In the low frequency region, the straight sloping line more towards imaginary axis and this indicates good capacitive behaviour of the electrode^49^.

The Nyquist plots in Fig. 7(b) were further analysed by a simulation method using the modified equivalent circuit that comprises of R_s_ (contact resistance), C_dl_ (double layer capacitance), R_ct_ (charge-transfer resistance), and W (Warburg component), as shown in inset of Fig. 7(b). The R_s_ represents the resistance of the electrolyte-electrode-current collector. The double layer capacitance (C_dl_), which is in parallel connection with R_ct_, represents the double layer capacitance between the ionic charge of the electrolytes and the electronic charges of the electrodes. Most importantly, due to the pseudo charge transfer resistance (R_ct_) a semicircle is appeared in the range of high frequency (inset of Fig. 7b). The calculated pseudo charge transfer resistance (R_ct_) of the Mn_3_O_4_ nanoparticles is 29 Ω cm^2^.

A comparative summary of different synthesis route and electrochemical properties of Mn_3_O_4_ nanostructures in aquous electrolyte is given in Table 2. From available literarure, observed capacitance values of Mn_3_O_4_ or Mn_3_O_4_-composite electrode synthesised via chemical/solvotherml route using reagent grade materials, vary from ~100 to 250 F g^−1^ for various electrolyte. Capacitance value of 125 F g^−1^ and cycle statbility of Mn_3_O_4_ NPs derived from waste Zn-C battery is compatible with literature data and future study will be conducted to improve the performance of Mn_3_O_4_ NPs following additional chemical treatment and/or synthesising composite materials with carbon materials.

**Table 2:** A comparative summary of different synthesis route and electrochemical properties of Mn_3_O_4_ nanostructures.

Material	Synthesis route	Electrochemical testing conditions	Capacitance (F g^−1^)	Reference
Mn_3_O_4_ film (reagent grade precursor)	Chemical bath deposition	Electrolyte: 1 M Na_2_SO_4_ Scan rate: 10 mV s^−1^ Electrodes: 3 electrode cell Working: Mn_3_O_4_ Counter: Pt Reference: saturated calomel	193	Dubal D. P. *et al*.^13^
Graphene/Mn_3_O_4_ composite (reagent grade precursor)	Solvothermal	Electrolyte: 1 M Na_2_SO_4_ Scan rate: 5 mV s^−1^ Electrodes: 3 electrode cell Working: Graphene/Mn_3_O_4_ Counter: Pt Reference: saturated calomel	225	Wu Y. *et al*.^50^
Graphene nanosheet/Mn_3_O_4_ composite (reagent grade precursor)	Chemical route	Electrolyte: 6 M KOH Scan rate: 5 mV s^−1^ Electrodes: 3 electrode cell Working: Graphene/Mn_3_O_4_ Counter: Pt foil Reference: saturated calomel	175	Wang B. *et al*.^51^
Graphene/Mn_3_O_4_ composite (reagent grade precursor)	Hydrothermal	Electrolyte: 1 M Na_2_SO_4_ Scan rate: 5 mV s^−1^ Electrodes: 3 electrode cell Working: Graphene/Mn_3_O_4_ Counter: Pt Reference: Ag/AgCl	114	Lee J. W. *et al*.^52^
Mn_3_O_4_ from Waste Zn-C battery	Thermal	Electrolyte: 0.6 M KOH Scan Rate: 10 mV s^−1^ Electrodes: 3 electrode cell Working: Mn_3_O_4_ Counter: Pt Reference: saturated calomel	125	This study

## Conclusions

This study for the first time reports the reuse of waste Zn-C derived Mn_3_O_4_ NPs for supercapacitor application. Spherical and cubic shaped Mn_3_O_4_ particles were observed in micrograph and the polycrystalline nature were confirmed by SAED and XRD diffraction patterns. Formation of Mn_3_O_4_ NPs from waste Zn-C battery via thermal route was validated by XRD, Raman, FTIR and XPS analysis. Mn_3_O_4_ as electrode material for supercapacitor application showed good specific capacitance, cycle stability and good reversibility of electrochemical charge/discharge process. The specific capacitance 125 F g^−1^ at the scan rate of 10 mV s^−1^ and 117.56 F g^−1^ at current density of 0.8 Ag^−1^ in 0.6 M KOH electrolyte was achieved. The cycle stability retained 80% even after 2100 cycles. This study will not only step-forward the waste battery disposal but also expand the opportunity to revive waste battery as an effective material for energy storage application providing a great significance of environmental sustainability.

## Experimental

### Material and Method

**Figure 8:**
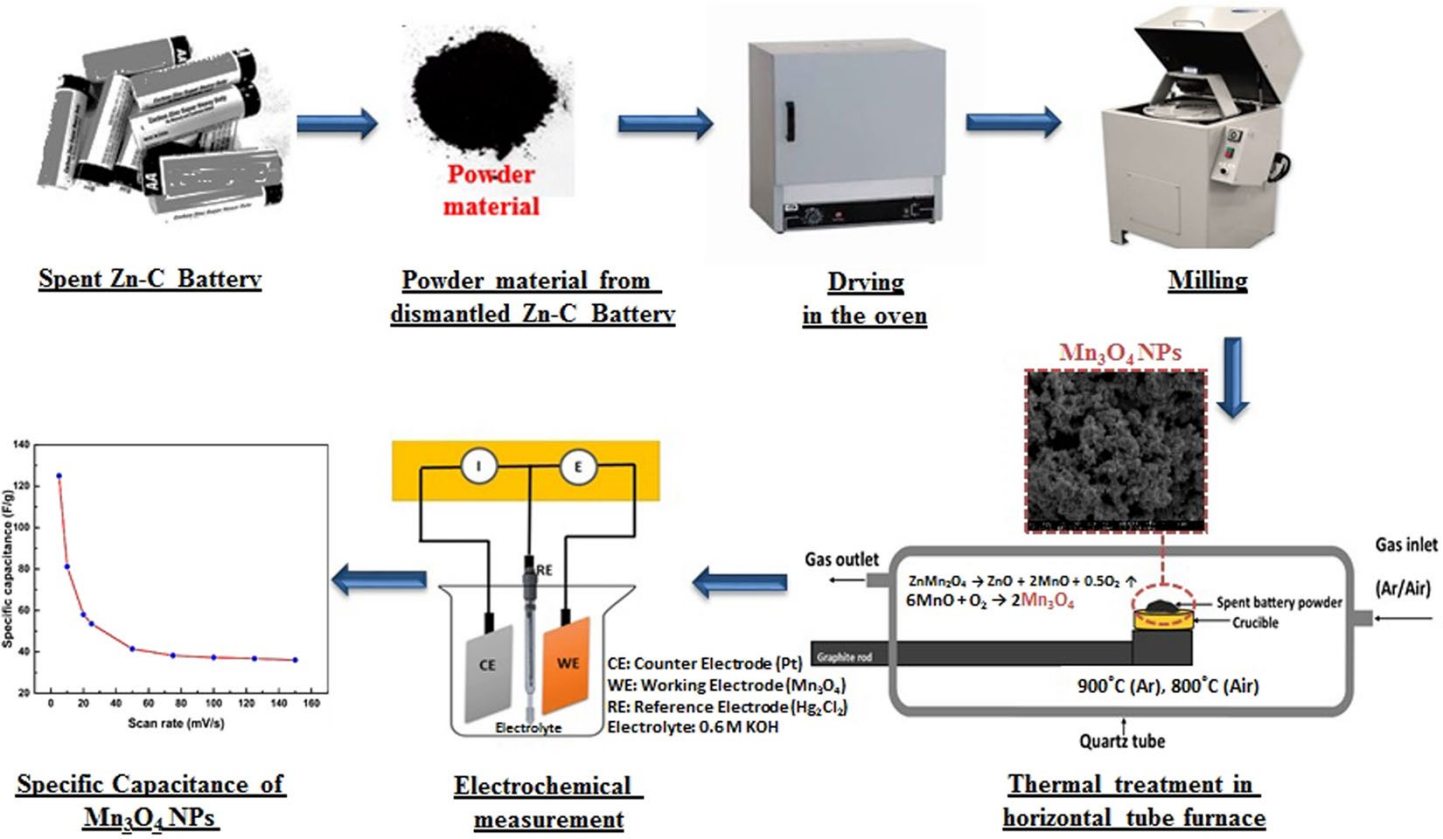
Schematic illustration of device fabrication process.

Waste Zn-C batteries were collected from UNSW Environmental Sustainability e-waste collection booth and manually dismantled. Powdered materials, after separating the Zn casing, outer metal shell, sealings, carbon rod etc. were used as raw materials to synthesise Mn_3_O_4_ NPs which finally tested for electrochemical performance. Dismantled fraction including schematic construction of Zn-C battery is shown and discussed in detail in our previous study^33^. In general, the powdered material in Zn-C battery contains manganese dioxide (MnO_2_) and is act as cathode which is wetted with electrolyte. Zinc chloride (ZnCl_2_) paste is used in heavy duty Zn-C battery as electrolyte to ensure continuity in the flow of current between cathode and anode. Spent Zn-C battery powder was dried in the oven at 90 °C for 2 h to remove moisture. The dried powder was pulverized in a ball milling machine to reduce agglomeration and later used for thermal study. A horizontal tube furnace composed of a quartz tube (length: 150 mm and diameter: 45 mm), gas flow system and graphite rod to hold the sample, was used for this experiment. The flow diagram including schematic illustration of experimental set-up is shown in Fig. 8. A graphite rod was used to carry the sample inside the furnace where temperature was 900 °C. Ceramic crucible loaded with the dried battery powder was placed on the graphite rod which was then pushed into the furnace and kept there for 1 hour under argon atmosphere. The greenish solid residue was pulled out and cooled down after 1 h and again kept into the furnace under air atmosphere for another 1 h at 800 °C and used for analysis and as active material for electrode fabrication. A schematic flow diagram of different steps to produce Mn_3_O_4_ and electrode fabrication steps are shown in Fig. 8.

### Characterization methods

Axios Advanced WDXRF with Rh end-window tube “Superq Software”, X-ray fluorescence spectroscopy (XRF) was used to determine the chemical composition of waste battery mixture. Simultaneous thermal analyser STA 8000, PerkinElmer, Thermogravimetric (TGA) analysis was conducted using under nitrogen atmosphere with a heating rate of 20 °C/min from room temperature to 1200 °C. PANalytical X’Pert Pro multipurpose X-ray diffraction (XRD) using CuKa radiation (λ = 1.54 Å) was used to identify phases and crystal structure. Xpert high score plus software was used for phase identification by XRD. Bruker X flash, Energy-dispersive X-ray Spectroscopy (EDS) 5010 was also used to determine elemental compositions. The morphology and microstructure were observed by JEOL 7001 F, field emission scanning electron microscope (FE-SEM), transmission electron microscope (TEM) of JEOL-1400 along with selected area electron diffraction (SAED). For EDS and microscopic analysis, sample powders were coated with platinum. Thermo Scientific ESCALAB250Xi using mono-chromated Al K alpha X-ray source with spot size of 500 micrometres, X-Ray Photoelectron Spectroscopy (XPS) analysis, FTIR, Spectrum 100 PerkinElmer, Fourier transform infrared spectroscopy measurement with a spectral range of 500 cm^−1^ to 4000 cm^−1^ using KBR and Renishaw inVia coupled with a microscope, Raman spectrometer using 514 nm argon ion lasers were used to characterize the nanoparticles derived from spent Zn-C battery. Contact angle goniometry (Kruss DSA 100 easy drop) was used measure contact angle of water droplet by the sessile drop method at normal temperature.

### Electrochemical measurements

A supercapacitor device was fabricated by drop-casting 10 µL of a conductive ink on a platinum electrode (inner diameter: 3 mm and outer diameter: 6 mm). Before drop casting, Platinum electrode was polished sequentially with different grades of emery paper, followed by cleaning with soap solution and washing with DI water and with acetone and then dried. The conductive ink was made up by adding 10 mg of active materials to be tested to 200 µL methanol and 25 µl of Nafion from Sigma Aldrich (2.5 wt.% in a mixture of lower aliphatic alcohols and water). The prepared suspension was sealed properly, sonicated for 30 min and finally magnetically-stirred for overnight. Before starting the electrochemical analysis, the working electrode was conditioned in the electrolyte for 3 h. Cyclic voltammetry (CV), galvanostatic charge–discharge (GCD) and electrochemical impedance spectroscopy (EIS) were performed by using a Biologic VSP-300 electrochemical workstation at room temperature.

The electrochemical studies were carried out following a standard three-electrode system containing the conductive ink coated platinum electrode as working electrode, Pt wire as a counter electrode, and saturated calomel (Hg_2_Cl_2_) electrode as a reference electrode. Aqueous solution of 0.6 M KOH was used as electrolyte. CV and GCD techniques were carried out within the potential range of 0 to +0.6 V at different scan rates (5, 10, 20, 25, 50, 75, 100, 125, and 150 mV s^−1^) and current densities (0.8, 1.2, 1.6, 2.0, 2.4, 2.8, 3.2 and 3.6 Ag^−1^), respectively. CV and GCD data were considered to evaluate the performance of active material derived from spent Zn-C battery for supercapacitor application. Electrochemical impedance spectroscopy (EIS) measurements were achieved under open circuit voltage in an alternating current frequency range of 10 kHz–100 MHz with an excitation signal of 0.6 V.

## Supplementary information

Manganese oxide synthesized from spent Zn-C battery for supercapacitor electrode application
