# Supplementary material for: Manganese oxide synthesized from spent Zn-C battery for supercapacitor electrode application
Source: Sci Rep. 2019 Jun 20;9:8982. doi: 10.1038/s41598-019-44778-z (PMC6586686; doi:10.1038/s41598-019-44778-z)
Supplement: Supplementary file 1 — Manganese oxide synthesized from spent Zn-C battery for supercapacitor electrode application [file 41598_2019_44778_MOESM1_ESM.docx]

**Supplementary Information**

**Manganese oxide synthesized from spent Zn-C battery for supercapacitor electrode application**

**Rifat Farzana** ^1^***, Kamrul Hassan**^1^ **and Veena Sahajwalla**^1^

**^1^** Centre for Sustainable Materials Research and Technology (SMaRT@UNSW),

School of Materials Science and Engineering, UNSW Sydney, Australia NSW 2052.

**Corresponding author**

Rifat Farzana ([r.farzana@unsw.edu.au](mailto:r.farzana@unsw.edu.au))

TGA Analysis of waste Zn-C battery powder under Nitrogen atmosphere at a heating rate of 20 ˚C min from room temperature to 1200 ˚C.


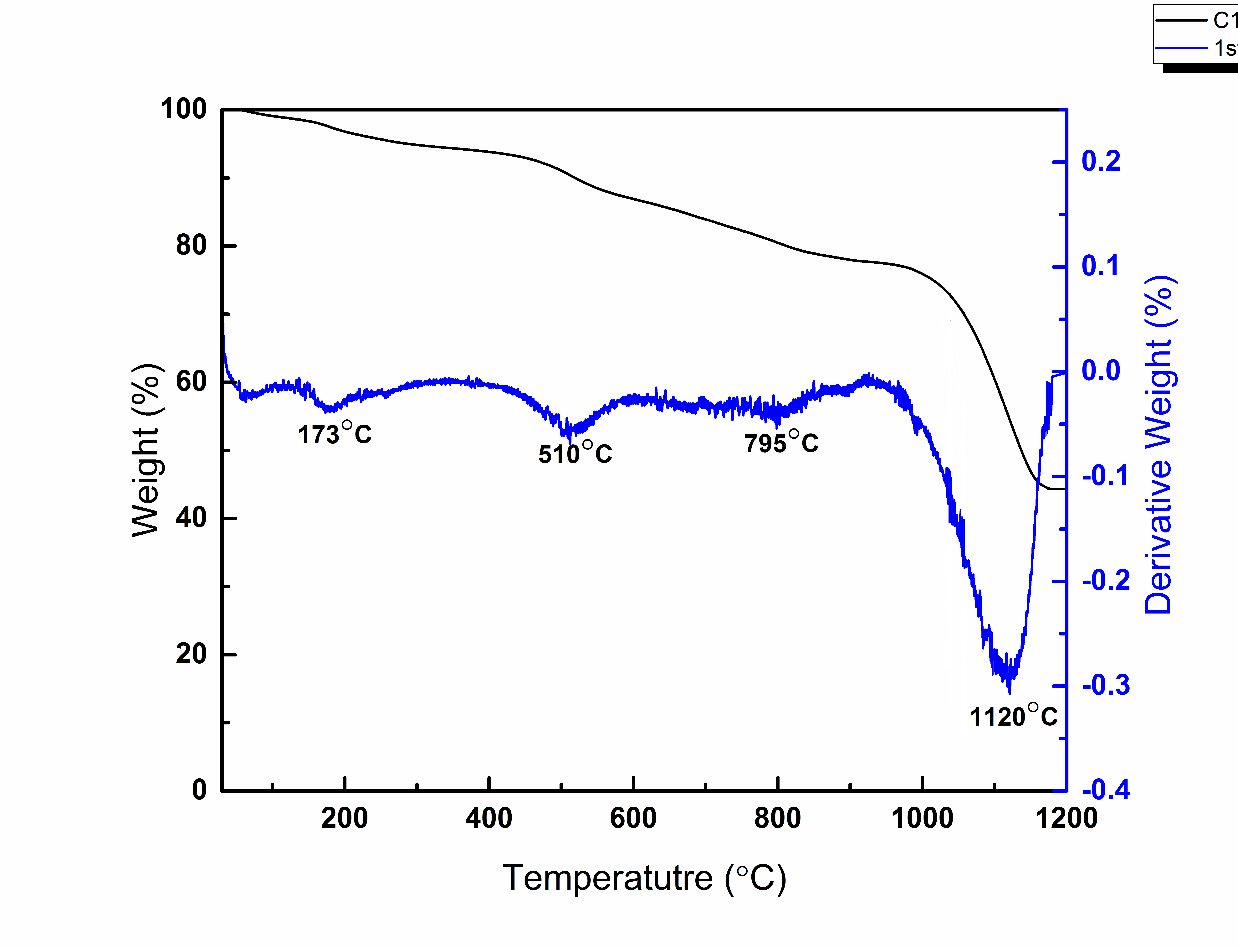


**Figure 1:** TGA analysis of waste Zn-C battery powder.

Contact angle of glass and Mn_3_O_4_/glass using the sessile drop method with distilled deionized water droplets (about 3 µl) at room temperature.


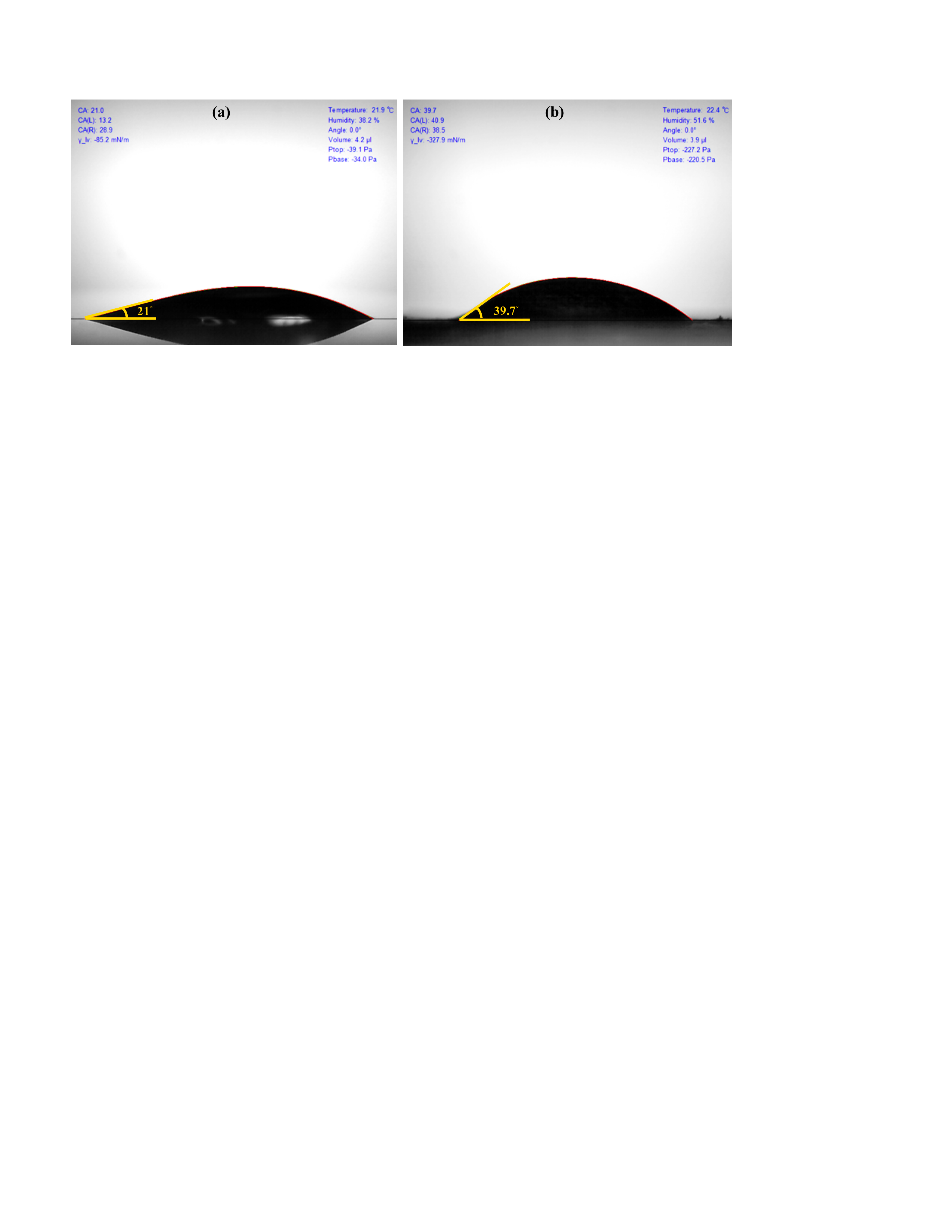


**Figure 2.** Contact angle images of (a) glass, and (b) Mn_3_O_4_/glass.

TGA Analysis of synthesised Mn_3_O_4_ NPs under Nitrogen atmosphere at a heating rate of 20 ˚C/ min from room temperature to 1200 ˚C.





**Figure 3:** TGA analysis of synthesised Mn_3_O_4_ NPs to show the thermal stability.

Quantitative ohmic/IR drop in Galvanostatic charge-discharge curves at different current densities is shown below.


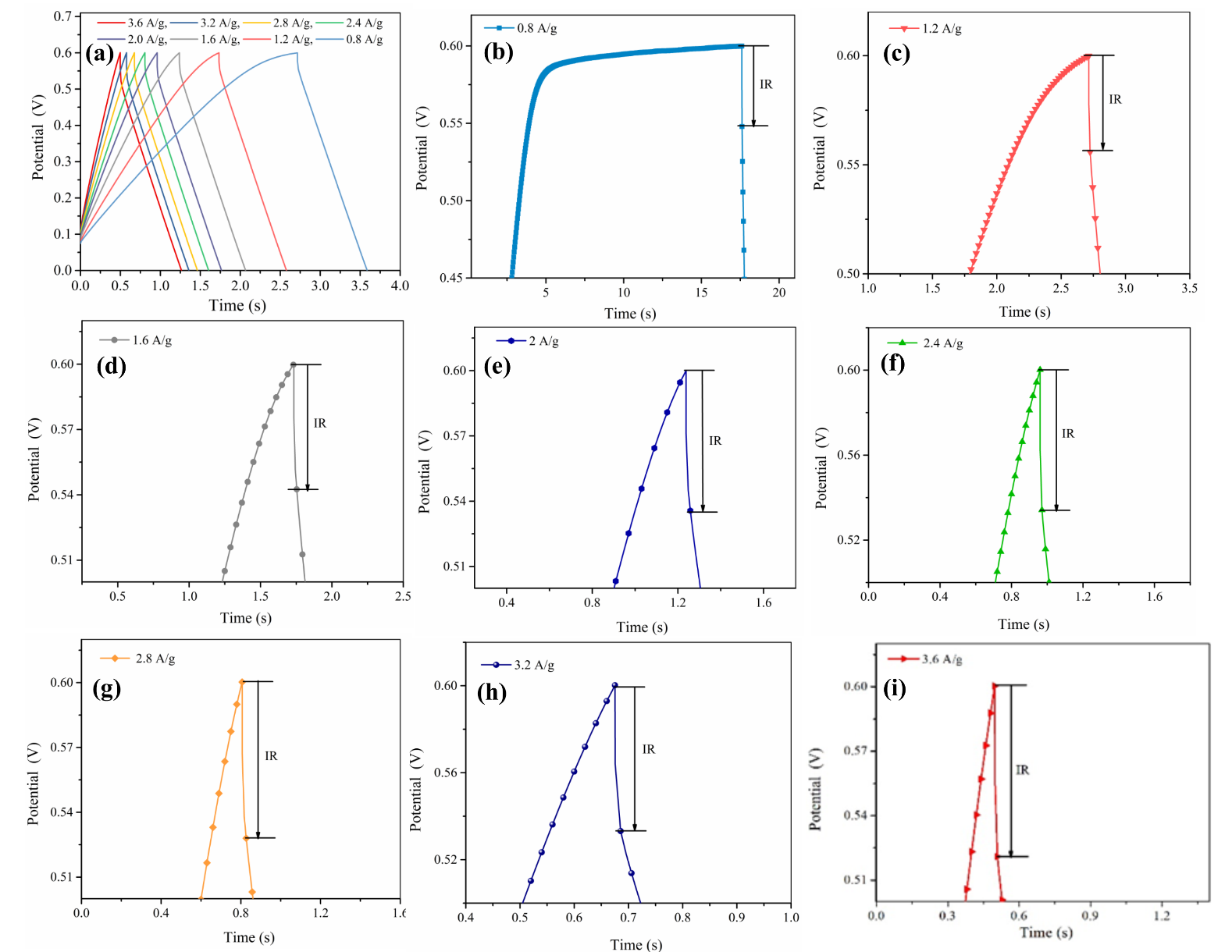


**Figure 4:** (a) Galvanostatic charge-discharge curves and IR drop in Galvanostatic charge-discharge curves at different current densities, (b) 0.8 A/g, (c) 1.2 A/g, (d) 1.6 A/g, (e) 2 A/g, (f) 2.4 A/g, (g) 2.8 A/g, (h) 3.2 A/g, (i) 3.6 A/g.
